# Supplementary material for: Identification of QTLs for Heat Tolerance at the Flowering Stage Using Chromosome Segment Substitution Lines in Rice
Source: Genes (Basel). 2022 Nov 30;13(12):2248. doi: 10.3390/genes13122248 (PMC9777623; doi:10.3390/genes13122248)
Supplement: Supplementary file 1 [file genes-13-02248-s001.zip › genes-2010436-supplementary.pdf]

Supplementary data

Additional supplementary data for this article can be found online:

Figure S1. The distribution of the introgressed segments according to genetic length (cM).

Figure S2. Frequency distribution for seed-setting rate of rice at natural environment (**A**,**C**) high temperature environment (**B**,**D**) in the CSSLs population in 2014 and 2015, respectively.

Additional files

Additional file 1:

Figure S1.

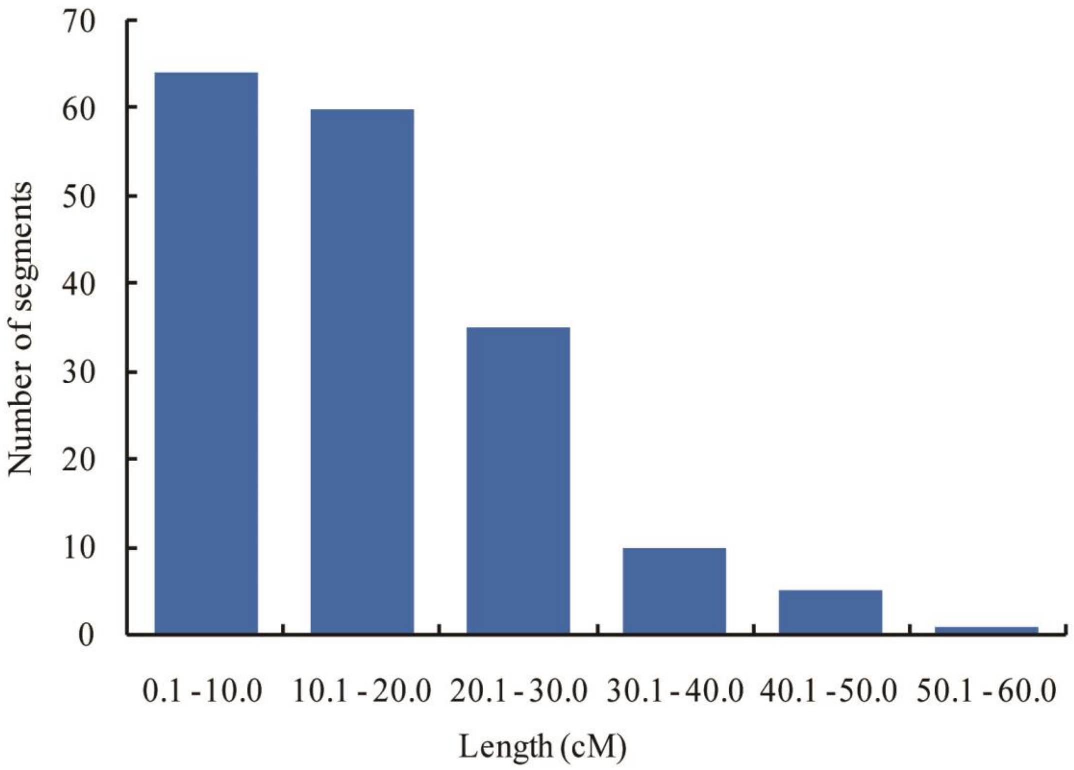

Figure S1. The distribution of the introgressed segments according to genetic length (cM).

Additional file 2:

Figure S2.

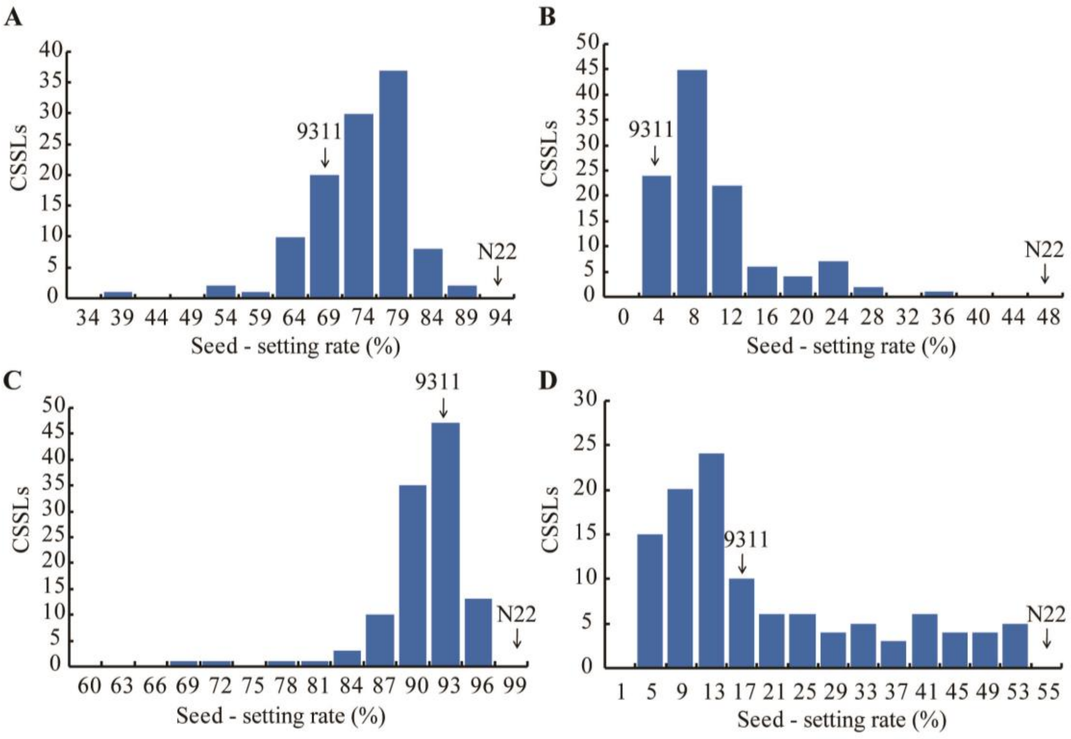

Figure S2. Frequency distribution for seed-setting rate of rice at natural environment (**A**,**C**) high temperature environment (**B**,**D**) in the CSSLs population in 2014 and 2015, respectively.
